# Risk Factors for *Pneumocystis jirovecii* Pneumonia in Rheumatoid Arthritis: The Protective Potential of Salazosulfapyridine

**DOI:** 10.1111/1756-185X.70318

**Published:** 2025-06-10

**Authors:** Yuki Hara, Takuro Nii, Takanori Matsuki, Kazuyuki Tsujino, Keisuke Miki, Akira Miyama, Koichiro Takahi, Hiroshi Kida

**Affiliations:** ^1^ Department of Respiratory Medicine NHO Osaka Toneyama Medical Center Toyonaka Japan; ^2^ Department of Orthopedic Surgery NHO Osaka Toneyama Medical Center Toyonaka Japan

**Keywords:** interstitial pneumonia, *pneumocystis jirovecii* pneumonia, protective factor, rheumatoid arthritis, risk factor, salazosulfapyridine

## Abstract

**Objectives:**

The aim of this study was to identify the risk factors for *Pneumocystis jirovecii* pneumonia (PCP) in patients with rheumatoid arthritis (RA) and to recommend appropriate disease‐modifying antirheumatic drug (DMARD) selection for those at high risk of developing PCP.

**Methods:**

We conducted a retrospective review of patients with RA who were treated with methotrexate or biologic and targeted synthetic DMARDs (b/tsDMARDs). Patients with no chest computed tomography data or those receiving prophylactic sulfamethoxazole–trimethoprim, atovaquone, or inhaled pentamidine were excluded.

**Results:**

Among 554 patients who met the inclusion and exclusion criteria, 16 developed PCP. Multivariate logistic analysis revealed interstitial pneumonia as a significant risk factor for PCP (odds ratio: 4.53, 95% confidence interval: 1.51–13.12), whereas salazosulfapyridine (SASP) use was associated with a reduced risk (odds ratio: 0.11, 95% confidence interval: 0.00–0.81). A Kaplan–Meier analysis comparing the cumulative incidence of PCP between propensity score‐matched SASP users and nonusers further demonstrated that SASP use reduced the risk of developing PCP.

**Conclusion:**

Patients with RA complicated by interstitial pneumonia are at a higher risk of developing PCP. Although methotrexate and b/tsDMARDs do not increase the risk of developing PCP, SASP may potentially reduce the risk. A prospective study is warranted to investigate the efficacy and safety of SASP in patients with RA at high risk for PCP.

**Summary**

- Interstitial pneumonia, but not chronic obstructive pulmonary disease (COPD), bronchiectasis, or lung cancer, is a risk factor for PCP.
- MTX or b/tsDMARDs are not PCP risk factors, whereas SASP is a protective factor.
- SASP is a good treatment option for patients with RA with interstitial pneumonia.

## 1 Introduction

*Pneumocystis jirovecii* pneumonia (PCP) is a life‐threatening opportunistic infection that primarily affects immunocompromised individuals, including patients with rheumatoid arthritis (RA) undergoing immunosuppressive therapy. With the increasing use of disease‐modifying antirheumatic drugs (DMARDs), particularly biologic and targeted synthetic DMARDs (b/tsDMARDs), concerns regarding PCP risk in patients with RA have escalated. Identifying risk factors for PCP and optimizing DMARD selection are essential for preventing this serious complication.

Previous studies have suggested that advanced age, preexisting pulmonary diseases, and steroid use may predispose patients with RA to PCP [1, 2, 3, 4]. Nevertheless, these studies have been limited by small sample sizes and heterogeneous patient populations. Moreover, although methotrexate (MTX) and b/tsDMARDs are widely used for RA treatment, their association with increased PCP risk remains unclear. Conversely, certain conventional synthetic DMARDs (csDMARDs), such as salazosulfapyridine (SASP), have been hypothesized to modulate immune responses in a manner that may influence the risk of infection [5].

In this study, our aim was to identify the risk factors for PCP in patients with RA and investigate the potential protective effect of SASP in those at high risk. Understanding these relationships could help guide safer therapeutic strategies and inform prophylactic measures against PCP in RA management.

## 2 Methods

### 2.1 Patients

**FIGURE 1:**
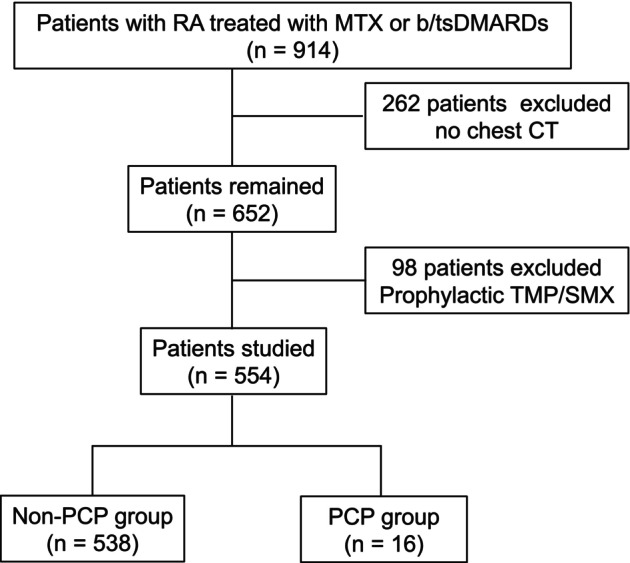
Flow chart of patient selection. Patients with RA treated with MTX or b/tsDMARDs were included (*n* = 914). Among them, 652 underwent chest CT. After excluding those receiving TMP/SMX prophylaxis (*n* = 98), the final study population consisted of 554 patients. Among them, 16 patients developed PCP. b/tsDMARDs, biologic and targeted synthetic disease‐modifying antirheumatic drugs; CT, computed tomography; MTX, methotrexate; PCP, *Pneumocystis jirovecii* pneumonia; RA, rheumatoid arthritis; TMP/SMX, sulfamethoxazole–trimethoprim.

We extracted the data from 914 patients with RA from electronic medical records who had been followed up at Osaka Toneyama Medical Center for at least 6 months between January 2012 and February 2025 and had a history of receiving at least one of MTX or b/tsDMARDs. After excluding 262 patients who had no chest computed tomography (CT) data and 98 patients who had received prophylactic sulfamethoxazole–trimethoprim (TMP/SMX), atovaquone, or inhaled pentamidine, 554 patients were included in this study (Figure 1). All of these patients fulfilled either the 1987 American College of Rheumatology (ACR) criteria or the 2010 ACR/European League Against Rheumatism criteria.

### 2.2 PCP Diagnostic Criteria

Patients were diagnosed if patients exhibited clinical symptoms consistent with its progression or demonstrated acute‐onset diffuse interstitial lung disease on chest plain CT, in addition to a positive β‐d‐glucan test or detection of *P. jirovecii* in sputum or bronchoalveolar lavage fluid via PCR. Additionally, patients must have received a therapeutic dose of TMP/SMX, atovaquone, or inhaled pentamidine during hospitalization.

### 2.3 Study Design

We conducted a retrospective review of the electronic medical records of all 554 patients, including diagnoses, prescriptions, and laboratory test results. All chest CT images were also evaluated. To identify the risk factors for PCP, we collected the following clinical parameters: gender, age, body mass index (BMI), duration of follow‐up, positivity for rheumatoid factor (RF), anti‐cyclic citrullinated peptide (ACPA), coexistence of interstitial pneumonia, chronic obstructive pulmonary disease (COPD), bronchiectasis, and lung cancer, as well as the use and dosages of MTX, b/tsDMARDs, csDMARDs other than MTX, and prednisolone (PSL). This study was conducted in accordance with the Declaration of Helsinki, and the research protocol was approved by the Institutional Review Board of the National Hospital Organization of Osaka Toneyama Medical Center (approval number TNH‐R‐2020056). The requirement for informed consent was waived due to the retrospective nature of the analysis. The opt‐out recruitment method was applied to provide an opportunity to decline participation for all patients.

### 2.4 Statistical Analysis

Continuous data are presented as median and interquartile range (IQR), whereas categorical data are presented as frequencies and percentages. Comparisons of continuous variables between non‐PCP and PCP groups were performed using the Wilcoxon rank‐sum test, whereas differences in categorical variables were evaluated using Fisher's exact test. To identify the risk factors for PCP, Firth‐corrected logistic regression analyses were conducted. Variables were selected based on previous reports [1, 2, 3, 4, 5, 6, 7], and their clinical relevance, with efforts made to exclude correlated variables whenever possible. The time to the incidence of PCP was compared using the Kaplan–Meier analysis. The index date was defined as the date of the first SASP use for SASP users and a randomly chosen clinic visit for SASP nonusers. As this was a retrospective study, each patient's random enrollment was defined as the date of RA onset and/or a date after January 2012, and age was determined based on that enrollment date. To balance background characteristics, propensity score matching was conducted to ensure that the standard mean difference (SMD) between the two groups was < 0.1. A two‐sided *p* value of < 0.05 was considered statistically significant. All statistical analyses were performed using R (version 4.4.1) and Prism 9.

## 3 Results

Of the 554 patients, 16 were diagnosed with PCP (PCP group), and the remaining 538 comprised the non‐PCP group (Figure 1). Table 1 presents the comparative clinical characteristics between the two groups. Patients in the non‐PCP group were younger (median age 65.6 years, IQR 58.0–75.0) than those in the PCP group (median age 71.3 years, IQR 68.0–74.0); although this difference was not statistically significant (*p* = 0.082). No significant differences were observed in gender, BMI, or positivity rates for RF and ACPA (Table 1). We also compared the prevalence of pulmonary comorbidities and found no significant differences for COPD (11.5% vs. 12.5%; *p* = 0.706), bronchiectasis (9.5% vs. 6.3%; *p* > 0.999), or lung cancer (6.0% vs. 6.3%; *p* > 0.999). However, interstitial pneumonia was significantly more common in the PCP group (43.8%) than in the non‐PCP group (11.7%; *p* = 0.002). We next evaluated the RA treatment regimens and found no significant differences between the non‐PCP and PCP groups in MTX usage (95.9% vs. 93.8%, *p* = 0.497) or dosage (8.8 mg/week vs. 8.4 mg/week, *p* = 0.598). Similarly, there were no significant differences in the use of b/tsDMARDs (47.2% vs. 43.8%, *p* = 0.806) or their concomitant use with MTX (43.1% vs. 37.5%, *p* = 0.800). Moreover, the PSL usage rate (11.5% vs. 18.8%, *p* = 0.418) and dosage (3.7 mg vs. 3.4 mg, *p* = 0.718) showed no significant differences. In particular, none of the patients in the PCP group received SASP, and the difference in SASP usage was statistically significant (25.3% vs. 0.0%, *p* = 0.016), with no significant differences observed for other csDMARDs.

**TABLE 1:** Comparative clinical characteristics between patients with and without PCP.

	Non‐PCP (*n* = 538)	PCP (*n* = 16)	*p*
Female	399 (74.2)	12 (75.0)	> 0.999
Age (years)	65.6 (58.0–75.0)	71.3 (68.0–74.0)	0.082
BMI (kg/m^2^)	21.7 (19.0–24.0)	21.4 (18.4–24.3)	0.703
Observation period (months)	51.1 (20.0–73.0)	76.7 (46.0–109.0)	0.009
RF‐positive	519 of 525 (98.9)	16 of 16 (100)	> 0.999
ACPA‐positive	365 of 438 (83.3)	15 of 16 (93.8)	0.488
RA stage I/II/III/IV	19.3/34.7/21.3/24.7	20.0/13.3/13.3/53.3	0.070
Pulmonary disease			
Interstitial pneumonia	63 (11.7)	7 (43.8)	0.002
COPD	62 (11.5)	2 (12.5)	0.706
Bronchiectasis	51 (9.5)	1 (6.3)	> 0.999
Lung cancer	32 (6.0)	1 (6.3)	> 0.999
Drug			
MTX	516 (95.9)	15 (93.8)	0.497
MTX dose (mg/week)	8.8 (6.0–12.0)	8.4 (6.0–10.5)	0.598
b/tsDMARD	254 (47.2)	7 (43.8)	0.806
MTX+ b/tsDMARD	232 (43.1)	6 (37.5)	0.800
Salazosulfapyridine	136 (25.3)	0 (0.0)	0.016
Iguratimod	96 (17.8)	2 (12.5)	0.750
Bucillamine	58 (10.8)	1 (6.3)	> 0.999
Tacrolimus	122 (22.7)	4 (25.0)	0.767
Mizoribine	13 (2.4)	0 (0.0)	> 0.999
Prednisolone	62 (11.5)	3 (18.8)	0.418
Prednisolone dose (mg/day)	3.7 (0.0–5.0)	3.4 (0.0–4.8)	0.718

*Note:* Data are presented as *n* (%) or median (interquartile range).

Abbreviations: ACPA, anti‐cyclic citrullinated peptide antibody; b/tsDMARD, biologic and targeted synthetic antirheumatic drug; BMI, body mass index; COPD, chronic obstructive pulmonary disease; MTX, methotrexate; PCP, *Pneumocystis jirovecii* pneumonia; RA, rheumatoid arthritis; RF, rheumatoid factor.

**FIGURE 2:**
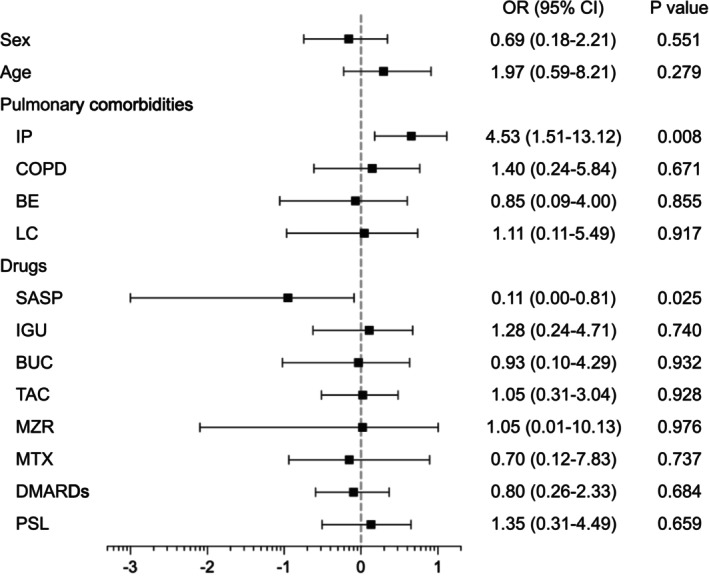
Multivariable logistic analysis of risk factors for PCP in patients with RA. BE, bronchiectasis; BUC, bucillamine; COPD, chronic obstructive pulmonary disease; DMARDs, disease‐modifying antirheumatic drugs; IGU, iguratimod; IP, interstitial pneumonia; LC, lung cancer; OR, odds ratio; MZR, mizoribine; MTX, methotrexate; PSL, prednisolone; SASP, salazosulfapyridine; TAC, tacrolimus.

We next performed a multivariate analysis to identify the independent risk factors for PCP (Figure 2), wherein the variables, such as age, gender, existing pulmonary comorbidities (interstitial pneumonia, COPD, bronchiectasis, and lung cancer), and RA medications were considered. Neither gender (*p* = 0.551) nor age (*p* = 0.279) was significantly associated with PCP risk. Among pulmonary comorbidities, only interstitial pneumonia was significantly associated with an increased risk of developing PCP (*p* = 0.008), whereas COPD (*p* = 0.671), bronchiectasis (*p* = 0.855), and lung cancer (*p* = 0.917) were not. Regarding RA medications, SASP use was significantly associated with a reduced risk of developing PCP (*p* = 0.025), whereas the use of MTX, b/tsDMARDs, PSL, and other medications demonstrated no significant associations.

**FIGURE 3:**
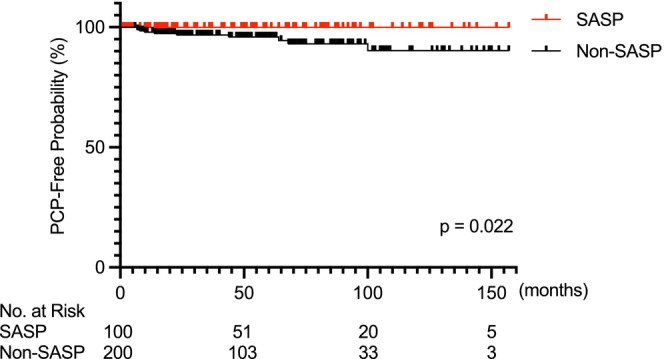
Comparison of PCP incidence in patients with RA with and without SASP treatment. In the comparison between the SASP group and non‐SASP group, the incidence of PCP was significantly lower in the SASP group (log‐rank test). PCP, *Pneumocystis jirovecii* pneumonia; RA, rheumatoid arthritis; SASP, salazosulfapyridine.

To clarify the time‐dependent effect of SASP in suppressing PCP development, we compared the incidence of PCP between the SASP nonuser group (*n* = 418) and the SASP user group (*n* = 136) using a Kaplan–Meier analysis. As shown in Table 2, baseline differences were observed between these groups, which were well balanced after propensity score matching between the non‐SASP user group (*n* = 200) and the SASP user group (*n* = 100). After matching, the Kaplan–Meier analysis revealed a significantly lower incidence of PCP in the SASP group. The estimated 10‐year incidence of PCP in the non‐SASP group was 9.8% compared to 0.0% in the SASP group (*p* = 0.022) (Figure 3).

**TABLE 2:** Comparative clinical characteristics between patients with and without SASP use before and after propensity score matching.

Before PS matching	Non‐SASP (*n* = 418)	SASP (*n* = 136)	SMD
Female	313 (74.9)	98 (72.1)	0.064
Age (years)	68.0 (58.0–74.0)	67.0 (59.0–75.0)	0.061
BMI (kg/m^2^)	21.5 (19.0–24.0)	20.9 (18.7–23.4)	0.046
Duration of taking SASP (months)	0.0 (0.0–0.0)	29.0 (11.0–71.3)	
Observation period (months)	45.0 (20.0–78.0)	62.5 (22.8–107.3)	0.397
RF‐positive	408 of 412 (99.0%)	127 of 129 (98.5%)	0.052
ACPA‐positive	227 of 337 (82.2%)	103 of 117 (88.0%)	0.513
Pulmonary disease			
Interstitial pneumonia	54 (12.9)	16 (11.8)	0.035
COPD	46 (11.0)	18 (13.2)	0.068
Bronchiectasis	37 (8.9)	15 (11.0)	0.073
Lung cancer	25 (6.0)	8 (5.9)	0.004
Drug			
MTX	405 (96.9)	126 (92.7)	0.191
MTX dose (mg/week)	10.0 (6.0–12.0)	8.0 (6.0–12.0)	0.184
b/tsDMARDs	203 (48.6)	58 (42.7)	0.119
PSL	51 (12.2)	14 (10.3)	0.060
PSL dose (mg/day)	5.0 (0.0–5.0)	5.0 (0.0–5.0)	0.078

After PS matching	Non‐SASP (*n* = 200)	SASP (*n* = 100)	SMD
Female	150 (75.0)	76 (76.0)	0.023
Age (years)	68.0 (59.0–74.0)	67.0 (59.0–76.0)	0.051
BMI (kg/m^2^)	21.5 (19.2–23.7)	20.9 (19.2–23.3)	0.028
Duration of taking SASP (months)	0.0 (0.0–0.0)	22.0 (11.0–55.3)	
Observation period (months)	52.5 (26.0–85.3)	50.5 (19.0–87.3)	0.027
RF‐positive	197 of 200 (98.5)	100 of 100 (100.0)	0.175
ACPA‐positive	176 of 200 (88.0)	88 of 100 (88.0)	0.000
Pulmonary disease			
Interstitial pneumonia	23 (11.5)	11 (11.0)	0.016
COPD	21 (10.5)	13 (13.0)	0.078
Bronchiectasis	24 (12.0)	12 (12.0)	0.000
Lung cancer	9 (4.5)	4 (4.0)	0.025
Drug			
MTX	192 (96.0)	96 (96.0)	0.000
MTX dose (mg/week)	10.0 (6.0–12.0)	8.0 (6.0–12.0)	0.065
b/tsDMARD	89 (44.5)	44 (43.0)	0.010
PSL	27 (13.5)	12 (12.0)	0.045
PSL dose (mg/day)	5.0 (0.0–5.3)	5.0 (0.0–5.0)	0.082

*Note:* Data are presented as *n* (%) or median (interquartile range).

Abbreviations: ACPA, anti‐citrullinated protein antibody; b/tsDMARD, biologic and targeted synthetic antirheumatic drug; BMI, body mass index; COPD, chronic obstructive pulmonary disease; MTX, methotrexate; PS, propensity score; PSL, prednisolone; RF, rheumatoid factor; SASP, salazosulfapyridine; SMD, standard mean difference.

## 4 Discussion

In this study, we first demonstrated that interstitial pneumonia, pulmonary comorbidity, was an independent risk factor for PCP among patients with RA treated with MTX or b/tsDMARDs. Unlike previous studies [3, 8, 9, 10], our cohort included patients who had not received biological agents, demonstrating that biological therapies have at most a minimal impact on the development of PCP. In our analysis, MTX was not identified as a risk factor for PCP. While prior studies have suggested that MTX may be associated with an increased risk of PCP [11, 12], some reports indicate that this risk may be more pronounced when MTX is used in the presence of other factors, such as advanced age or hypoalbuminemia [12, 13]. Although the current EULAR recommendations support prophylactic use of TMP/SMX in patients receiving corticosteroids, they do not specifically address MTX [14]. Our findings may therefore suggest that MTX use alone is not necessarily sufficient to increase the risk of PCP.

Moreover, by analyzing individual pulmonary comorbidities, we found that interstitial pneumonia, rather than COPD, bronchiectasis, or lung cancer, not only increases the risk for PCP‐related mortality as reported previously [2] but also elevates the risk of PCP onset.

Interestingly, we further demonstrated that SASP usage was an independent protective factor for PCP among patients with RA who had been treated with MTX or b/tsDMARDs. Mizushina et al. previously reported that none of the 61 patients receiving SASP developed PCP, whereas 10 of the 149 patients who did not receive SASP developed PCP [5]. Nonetheless, their study showed several differences in baseline patient characteristics, including the observation period, between the SASP and non‐SASP groups. To overcome the limitations of such a retrospective design, we used propensity score matching to balance these baseline characteristics and then performed a Kaplan–Meier analysis to compare the incidence of PCP between the SASP and non‐SASP groups.

In the pathogenesis of PCP, the pathogen itself causes direct tissue damage, but the host's excessive inflammatory response also plays a significant role [15, 16]. SASP is metabolized into 5‐aminosalicylic acid (5‐ASA) and sulfapyridine (SP) [17]. 5‐ASA suppresses the production of inflammatory cytokines such as IL‐1 and TNF‐α, whereas SP inhibits dihydropteroate synthase (DHPS), an enzyme essential for the growth of *P. jirovecii*. Similarly, SMX, a component of TMP/SMX, also targets DHPS, helping to prevent the development of PCP. Considering these mechanisms, SASP may help prevent the development of PCP by both reducing the production of inflammatory cytokines involved in its pathogenesis and directly inhibiting the growth of *P. jirovecii*. Although 98 patients taking TMP/SMX were excluded from our analysis, none of the patients on TMP/SMX developed PCP either. It remains unclear how SASP should be positioned in the treatment of RA, and further investigation, including the one on the mechanism of SASP's prophylactic effect against PCP, is warranted.

Although TMP/SMX is the first‐line drug for PCP prophylaxis in patients with RA, it is associated with a wide range of adverse effects, including gastrointestinal toxicity, rash, anemia, thrombocytopenia, neutropenia, hyperkalemia, and renal failure [18]. The discontinuation rate of TMP/SMX has been reported to be between 17%–61% [19, 20, 21]. Other preventive options include atovaquone and inhaled pentamidine; however, atovaquone is expensive, and inhaled pentamidine is often difficult to continue because of its complex administration method [22]. Therefore, adding SASP for patients with RA who are intolerant to TMP/SMX might be a beneficial strategy, as it has the potential to achieve both improved RA treatment and PCP prevention simultaneously.

In patients with RA complicated by interstitial pneumonia, MTX and TNF inhibitors are often avoided due to the risk of acute exacerbation of interstitial pneumonia, whereas abatacept is considered relatively safe [23, 24]. Our study demonstrates that, in these patients, it is also crucial to consider the risk of developing PCP. Adding SASP to abatacept—not only as a means to strengthen RA treatment but also for PCP prophylaxis—could be a promising new strategy. However, this must be validated through a prospective study to confirm its effectiveness and safety.

The limitations of this study include its retrospective design and single‐center nature. Our hospital treats a large number of patients with RA and respiratory comorbidities, which may have influenced the results. Furthermore, as a single‐center study, physicians' prescribing preferences could have affected the outcomes.

## Author Contributions

The authors take full responsibility for this article.

## Conflicts of Interest

The authors declare no conflicts of interest.

## Data Availability

The data underlying this article are available in the article.
